# Industrial Lead Poisoning

**Published:** 1908-05-23

**Authors:** 


### Industrial Lead Poisoning.

Yet another inquiry into the industrial aspects of lead-poisoning has been inaugurated at the instance of the Home Office, a committee having been appointed to consider the dangers attendant on the use of lead in the various branches of the manufacture of china and earthenware, and to report how far these can be obviated or lessened. Prior to the Act of 1895, which enjoined the compulsory notification of all cases of plumbism, the extent of the damage to health and life resulting from the industrial use of lead was only vaguely appreciated by the country at large; but the committee appointed in 1893 to inquire into the health of the workers in potteries laid bare the dimensions of the evil and initiated, with the Act above mentioned, a crusade for improvement which has since been actively pursued. This committee reported that "the general truth that the potteries occupation is one fraught with injury to health and life is beyond dispute." and that "the ill effects of the trade are referable to two chief causes—namely, dust, and the poison of lead." The dangers of a dusty atmosphere are not, of course, by any means limited to the dust of potteries; indeed, the current teaching of the schools would lead one to infer that respiratory diseases due to the perpetual inhalation of dust are the special prerogative of other industries, particularly masonry, coal-mining, and knife-grinding. Yet, contrary to general belief, plumbism in potteries is a poor second to respiratory diseases as a cause of death. None the less the damage done by lead fully justifies the persistent efforts which have been and are being made to minimise its deleterious influences.

The use of lead in pottery is, of course, related to the stage of glazing by which the porous earthenware is made watertight, the addition of lead to the silicious materials employed rendering the glaze more easily fusible, and thus more readily incorporated with the pottery to which it is applied. The committee of 1893, failing to see any immediate prospect of leadless glazes becoming universally applicable to pottery manufacture, recommended that the dangers associated with the use of raw lead might be much minimised by a process known as "fritting." "Fritting" the lead implies a mixing of it with the other ingredients of the glaze and fusing them together into a kind of rough glass which is subsequently ground; by this means the lead becomes less soluble, and thus less potent for harm than when added in a raw state. In spite, however, of these recommendations, a severe outbreak of lead-poisoning in 1897 led to more stringent regulations, prohibiting the employment of persons under fifteen years of age in the dangerous processes, ordering a monthly examination of all women and young persons working in lead, and providing for the more effectual removal of dust and the better enforcement of cleanliness. The effect was immediate, the number of cases of lead poisoning in the industry falling from 249 in 1899 to 106 in 1901.

In all matters connected with the security of industrial workers one of the most difficult things to combat is the ignorance or carelessness of the workers themselves. This is seen more particularly in the case of lead, chiefly perhaps because the penalties of carelessness are long postponed. Lead is a cumulative poison, and the careless workman who eats with unwashed hands may long continue to ingest the small doses to which he thus exposes himself before the fruits of his folly are exhibited. An experienced physician in the pottery district has stated that in the pottery trade, as compared with certain other industries, lead is very slow in producing serious effects. In his experience the average period of working in lead before serious lesions manifested themselves was eighteen years for females and twenty-two and a half years for males, though some individuals exhibited a remarkable susceptibility. It is little wonder that uninstructed people should laugh away a danger so remote. Even in bacteriological laboratories and post-mortem rooms, in dealings with virulent organisms capable of exacting swift retribution for acts of carelessness, one sees examples of the unwise contempt which is engendered by familiarity; and this, too, in the case of educated and expert workers. It is idle to expect more caution from the industrial population of the potteries, and thus the chief weapon against lead poisoning lies in stringent regulations. We may be sure that so long as lead continues to be used in the glazing of earthenware lead poisoning will continue to be an occasional accident of the trade. The new committee's terms of reference include the question of substitutes for lead, and we may hope that some such substitute, not commercially impracticable, will supply the simplest and only completely satisfactory solution of the problem.

